# Supplementary material for: Comparative outcomes of image-guided percutaneous catheterization versus direct visualization catheterization for peritoneal dialysis: A meta-analysis
Source: PLoS One. 2025 Jul 7;20(7):e0325600. doi: 10.1371/journal.pone.0325600 (PMC12233245; doi:10.1371/journal.pone.0325600)
Supplement: S1 Text — (DOCX) [file pone.0325600.s001.docx]

# PICO策略

1. Patients: patients with end-stage renal disease.
2. Intervention: peritoneal dialysis catheter using fluoroscopy or ultrasound guidance.
3. The study needs to meet the following criteria:

Inclusion criteria: (1) Patients with ESRD who underwent PD catheter placement; (2) Interventional group: patients received IGPC, comprising percutaneous puncture techniques utilising X-ray fluoroscopy or ultrasound guidance; (3) Control group: patients received DVC, comprising conventional open surgery or catheter placement techniques performed under laparoscopy; (4) At least one of the following outcomes was reported: peritonitis, tunnel infection, exit-site infection, catheter dysfunction, bleeding, catheter leakage, hernia, catheter removal, and one-year PD catheter survival. (5) Study design: RCT, prospective study, or retrospective study.

Exclusion criteria: (1) Other types of articles, including conferences, abstracts, yearbooks, case reports, journals, letters, reviews, meta-analyses, editorials, pharmaceutical interventions, animal studies, and protocols; (2) Not relevant; (3) Duplicate patient cohort; (4) Inability to extract data for meta-analysis.

# 2. 检索词

## 腹膜透析

Peritoneal Dialysis

Dialyses, Peritoneal

Dialysis, Peritoneal

Peritoneal Dialyses

Continuous Ambulatory Peritoneal Dialysis

Peritoneal Dialysis, Continuous Ambulatory

PD

CAPD

CCPD

APD

## **导管**

peritoneal dialysis catheter

peritoneal dialysis catheters

peritoneal catheter implantation

peritoneal catheter

indwelling catheter

catheter insertion

catheter implant

Catheterization

Catheter

Implantation

## **IGPC组**

**经皮**

Percutaneous

**透视**

Fluoroscopy

Fluoroscopic

Magnification, Radiographic

Magnifications, Radiographic

Radiographic Magnifications

**超声引导**

ultrasound-guided

## **DVC组**

**开放手术切开**

surgical insertion

Surgery

**腹膜切开**

Peritoneotomy

laparotomy

laparotomies

Minilaparotomy

Minilaparotomies

**腹腔镜**

Laparoscopes

Celioscopes

Celioscope

Laparoscope

Peritoneoscopes

Peritoneoscope

Laparoscopic

Laparoscopy

Laparoscopies

Peritoneoscopy

Peritoneoscopies

Celioscopy

Celioscopies

Surgical Procedures, Laparoscopic

Surgery, Laparoscopic

Laparoscopic Assisted Surgery

Laparoscopic Assisted Surgeries

Surgeries, Laparoscopic Assisted

Surgery, Laparoscopic Assisted

Laparoscopic Surgical Procedure

Laparoscopic Surgery

Laparoscopic Surgeries

Surgeries, Laparoscopic

Procedures, Laparoscopic Surgical

Surgical Procedure, Laparoscopic

Laparoscopic Surgical Procedures

Procedure, Laparoscopic Surgical

# 检索式

| **PubMed** | | |
| --- | --- | --- |
| NO | Query | Results |
| #1  Peritoneal Dialysis | (((((((((Peritoneal Dialysis[Title/Abstract]) OR (Dialyses, Peritoneal[Title/Abstract])) OR (Dialysis, Peritoneal[Title/Abstract])) OR (Peritoneal Dialyses[Title/Abstract])) OR (Continuous Ambulatory Peritoneal Dialysis[Title/Abstract])) OR (Peritoneal Dialysis, Continuous Ambulatory[Title/Abstract])) OR (PD[Title/Abstract])) OR (CAPD[Title/Abstract])) OR (CCPD[Title/Abstract])) OR (APD[Title/Abstract]) | **235,348** |
| #2  catheter | (((((((((peritoneal dialysis catheter[Title/Abstract]) OR (peritoneal dialysis catheters[Title/Abstract])) OR (peritoneal catheter implantation[Title/Abstract])) OR (peritoneal catheter[Title/Abstract])) OR (indwelling catheter[Title/Abstract])) OR (catheter insertion[Title/Abstract])) OR (catheter implant[Title/Abstract])) OR (Catheterization[Title/Abstract])) OR (Catheter[Title/Abstract])) OR (implantation[Title/Abstract]) | **411,120** |
| #3  IGPC | (((((((percutaneous[Title/Abstract])) OR (Fluoroscopic[Title/Abstract])) OR (fluoroscopy[Title/Abstract])) OR (Magnification, Radiographic[Title/Abstract])) OR (Magnifications, Radiographic[Title/Abstract])) OR (Radiographic Magnifications[Title/Abstract])) OR (ultrasound-guided[Title/Abstract]) | **237,818** |
| #4  DVC | (((((((((((((((((((((((((((((((((surgical insertion[Title/Abstract]) OR (Surgery[Title/Abstract])) OR (Laparoscopes[Title/Abstract])) OR (Celioscopes[Title/Abstract])) OR (Celioscope[Title/Abstract])) OR (Laparoscope[Title/Abstract])) OR (Peritoneoscopes[Title/Abstract])) OR (Peritoneoscope[Title/Abstract])) OR (Laparotomy[Title/Abstract])) OR (laparotomies[Title/Abstract])) OR (Minilaparotomy[Title/Abstract])) OR (Minilaparotomies[Title/Abstract])) OR (Laparoscopic[Title/Abstract])) OR (Peritoneotomy[Title/Abstract])) OR (Laparoscopy[Title/Abstract])) OR (Laparoscopies[Title/Abstract])) OR (Peritoneoscopy[Title/Abstract])) OR (Peritoneoscopies[Title/Abstract])) OR (Celioscopy[Title/Abstract])) OR (Celioscopies[Title/Abstract])) OR (Surgical Procedures, Laparoscopic[Title/Abstract])) OR (Surgery, Laparoscopic[Title/Abstract])) OR (Laparoscopic Assisted Surgery[Title/Abstract])) OR (Laparoscopic Assisted Surgeries[Title/Abstract])) OR (Surgeries, Laparoscopic Assisted[Title/Abstract])) OR (Surgery, Laparoscopic Assisted[Title/Abstract])) OR (Laparoscopic Surgical Procedure[Title/Abstract])) OR (Laparoscopic Surgery[Title/Abstract])) OR (Laparoscopic Surgeries[Title/Abstract])) OR (Surgeries, Laparoscopic[Title/Abstract])) OR (Procedures, Laparoscopic Surgical[Title/Abstract])) OR (Surgical Procedure, Laparoscopic[Title/Abstract])) OR (Laparoscopic Surgical Procedures[Title/Abstract])) OR (Procedure, Laparoscopic Surgical[Title/Abstract]) | 1,677,427 |
| #5 | #1 AND #2 AND #3 AND #4 | **159** |

| **Web of science** | | |
| --- | --- | --- |
| NO | Query | Results |
| #1  Peritoneal Dialysis | TS=(Peritoneal Dialysis OR Dialyses, Peritoneal OR Dialysis, Peritoneal OR Peritoneal Dialyses OR Continuous Ambulatory Peritoneal Dialysis OR Peritoneal Dialysis, Continuous Ambulatory OR PD OR CAPD OR CCPD OR APD) | **617,839** |
| #2  catheter | TS=(peritoneal dialysis catheter OR peritoneal dialysis catheters OR peritoneal catheter OR indwelling catheter OR Catheter OR catheter insertion OR catheter implant OR Catheterization OR implantation) | **1,484,619** |
| #3  IGPC | TS=(percutaneous OR Fluoroscopic OR fluoroscopy OR Magnification, Radiographic OR Magnifications, Radiographic OR Radiographic Magnifications OR ultrasound-guided) | **409,258** |
| #4  DVC | TS=(surgical insertion OR Surgery OR Laparoscopes OR Celioscopes OR Celioscope OR Celioscopes OR Laparoscope OR Peritoneoscopes OR Peritoneoscope OR Laparotomy OR laparotomies OR Minilaparotomy OR Minilaparotomies OR Laparoscopic OR Peritoneotomy OR Laparoscopy OR Laparoscopies OR Peritoneoscopy OR Peritoneoscopies OR Celioscopy OR Celioscopies OR Surgical Procedures, Laparoscopic OR Surgery, Laparoscopic OR Laparoscopic Assisted Surgery OR Laparoscopic Assisted Surgeries OR Surgeries, Laparoscopic Assisted OR Surgery, Laparoscopic Assisted OR Laparoscopic Surgical Procedure OR Laparoscopic Surgery OR Laparoscopic Surgeries OR Surgeries, Laparoscopic OR Procedures, Laparoscopic Surgical OR Surgical Procedure, Laparoscopic OR Laparoscopic Surgical Procedures Procedure, Laparoscopic Surgical) | **4,646,700** |
| #5 | #1 AND #2 AND #3 AND #4 | **407** |

| **Cochrane** | | |
| --- | --- | --- |
| NO | Query | Results |
| #1  Peritoneal Dialysis | (Peritoneal Dialysis OR Dialyses, Peritoneal OR Dialysis, Peritoneal OR Peritoneal Dialyses OR Continuous Ambulatory Peritoneal Dialysis OR Peritoneal Dialysis, Continuous Ambulatory OR PD OR CAPD OR CCPD OR APD):ab,ti,kw | **45,407** |
| #2  catheter | (peritoneal dialysis catheter OR peritoneal dialysis catheters OR peritoneal catheter OR indwelling catheter OR Catheter OR catheter insertion OR catheter implant OR Catheterization OR implantation):ab,ti,kw | **59,748** |
| #3  IGPC | (percutaneous OR Fluoroscopic OR fluoroscopy OR Magnification, Radiographic OR Magnifications, Radiographic OR Radiographic Magnifications OR ultrasound-guided):ab,ti,kw | **41,024** |
| #4  DVC | (surgical insertion OR Surgery OR Laparoscopes OR Celioscopes OR Celioscope OR Laparoscope OR Peritoneoscopes OR Peritoneoscope OR Laparotomy OR laparotomies OR Minilaparotomy OR Minilaparotomies OR Laparoscopic OR Peritoneotomy OR Laparoscopy OR Laparoscopies OR Peritoneoscopy OR Peritoneoscopies OR Celioscopy OR Celioscopies OR Surgical Procedures, Laparoscopic OR Surgery, Laparoscopic OR Laparoscopic Assisted Surgery OR Laparoscopic Assisted Surgeries OR Surgeries, Laparoscopic Assisted OR Surgery, Laparoscopic Assisted OR Laparoscopic Surgical Procedure OR Laparoscopic Surgery OR Laparoscopic Surgeries OR Surgeries, Laparoscopic OR Procedures, Laparoscopic Surgical OR Surgical Procedure, Laparoscopic OR Laparoscopic Surgical Procedures OR Procedure, Laparoscopic Surgical):ab,ti,kw | **301,147** |
| #5 | #1 AND #2 AND #3 AND #4 | **49** |

| **Embase** | | |
| --- | --- | --- |
| NO | Query | Results |
| #1  Peritoneal Dialysis | ‘Peritoneal Dialysis’/exp OR ‘Dialyses, Peritoneal’:ab,ti,kw OR ‘Dialysis, Peritoneal’:ab,ti,kw OR ‘Peritoneal Dialyses’:ab,ti,kw OR ‘Continuous Ambulatory Peritoneal Dialysis’:ab,ti,kw OR ‘Peritoneal Dialysis, Continuous Ambulatory’:ab,ti,kw OR ‘PD’:ab,ti,kw OR ‘CAPD’:ab,ti,kw OR ‘CCPD’:ab,ti,kw OR ‘APD’:ab,ti,kw | **392,962** |
| #2  catheter | ‘peritoneal dialysis catheter’/exp OR ‘peritoneal dialysis catheters’:ab,ti,kw OR ‘peritoneal catheter’:ab,ti,kw OR ‘indwelling catheter’:ab,ti,kw OR ‘Catheter’:ab,ti,kw OR ‘catheter insertion’:ab,ti,kw OR ‘catheter implant’:ab,ti,kw OR ‘Catheterization’:ab,ti,kw OR ‘implantation’:ab,ti,kw | **639,214** |
| #3  IGPC | ‘percutaneous’/exp OR ‘Fluoroscopic’:ab,ti,kw OR ‘fluoroscopy’:ab,ti,kw OR ‘Magnification, Radiographic’:ab,ti,kw OR ‘Magnifications, Radiographic’:ab,ti,kw OR ‘Radiographic Magnifications’:ab,ti,kw OR ‘ultrasound-guided’:ab,ti,kw | **368,676** |
| #3  DVC | ‘Laparoscopy’/exp OR ‘Laparoscopies’:ab,ti,kw OR ‘Peritoneoscopy’:ab,ti,kw OR ‘Peritoneoscopies’:ab,ti,kw OR ‘Celioscopy’:ab,ti,kw OR ‘Celioscopies’:ab,ti,kw OR ‘Surgical Procedures, Laparoscopic’:ab,ti,kw OR ‘Surgery, Laparoscopic’:ab,ti,kw OR ‘Laparoscopic Assisted Surgery’:ab,ti,kw OR ‘Laparoscopic Assisted Surgeries’:ab,ti,kw OR ‘Surgeries, Laparoscopic Assisted’:ab,ti,kw OR ‘Surgery, Laparoscopic Assisted’:ab,ti,kw OR ‘Laparoscopic Surgical Procedure’:ab,ti,kw OR ‘Laparoscopic Surgery’:ab,ti,kw OR ‘Laparoscopic Surgeries’:ab,ti,kw OR ‘Surgeries, Laparoscopic’:ab,ti,kw OR ‘Procedures, Laparoscopic Surgical’:ab,ti,kw OR ‘Surgical Procedure, Laparoscopic’:ab,ti,kw OR ‘Laparoscopic Surgical Procedures’:ab,ti,kw OR ‘Procedure, Laparoscopic Surgical’:ab,ti,kw OR ‘Peritoneotomy’:ab,ti,kw OR ‘Laparoscopic’:ab,ti,kw OR ‘Laparotomy’:ab,ti,kw OR ‘laparotomies’:ab,ti,kw OR ‘Minilaparotomy’:ab,ti,kw OR ‘Minilaparotomies’:ab,ti,kw OR ‘surgical insertion’:ab,ti,kw OR ‘surgery’:ab,ti,kw OR ‘Laparoscopes’:ab,ti,kw OR ‘Celioscopes’:ab,ti,kw OR ‘Celioscope’:ab,ti,kw OR ‘Laparoscope’:ab,ti,kw OR ‘Peritoneoscopes’:ab,ti,kw OR ‘Peritoneoscope’:ab,ti,kw | **2,363,878** |
| #5 | #1 AND #2 AND #3 AND #4 | **356** |

**检索时间范围：2000-01-01至 2024-07-16**
