# Supplementary material for: Comparative outcomes of image-guided percutaneous catheterization versus direct visualization catheterization for peritoneal dialysis: A meta-analysis
Source: PLoS One. 2025 Jul 7;20(7):e0325600. doi: 10.1371/journal.pone.0325600 (PMC12233245; doi:10.1371/journal.pone.0325600)
Supplement: S3 Text — (DOCX) [file pone.0325600.s003.docx]

| **Study** | **IGPC** | **DVC** | |
| --- | --- | --- | --- |
|  |  | **Laparoscopic** | **Open surgery** |
| Rosenthal et al.2008 | Laparoscopic insertion of a PD catheter is performed under general anesthesia. The abdomen is insufflated and several small incisions are made lateral to the umbilicus. A needle is inserted with the establishment of pneumoperitoneum. A 5-mm trocar is inserted under direct visualization and a second 5-mm trocar is inserted just on the left lateral midline below the umbilicus, aiming towards the pelvis. The catheter is then threaded through the trocar and the cuff is pulled back so that it lays within the abdominal wall musculature. The catheter is then tunneled subcutaneously so it exits at the site of the original 5-mm trocar placement. The catheter is flushed to assess appropriate placement, and the skin sites are closed with interrupted suture.  **Hospitalization time(day):25.7± 17.5** | Laparoscopic insertion of a PD catheter is performed under general anesthesia. The abdomen is insufflated and several small incisions are made lateral to the umbilicus. A needle is inserted with the establishment of pneumoperitoneum. A 5-mm trocar is inserted under direct visualization and a second 5-mm trocar is inserted just on the left lateral midline below the umbilicus, aiming towards the pelvis. The catheter is then threaded through the trocar and the cuff is pulled back so that it lays within the abdominal wall musculature. The catheter is then tunneled subcutaneously so it exits at the site of the original 5-mm trocar placement. The catheter is flushed to assess appropriate placement, and the skin sites are closed with interrupted suture.  **Hospitalization time(day):28.0± 14.3** | Conventionally, a PD catheter is placed under local or general anesthesia through a 5-mm incision traversing the skin, subcutaneous tissue, and anterior rectus sheath. A small incision is made to the peritoneal cavity and the catheter threaded on a stiffening stylet and introduced deeply into the true pelvis, and the purse-string suture was closed snugly around the tube. The cuff was placed between the posterior rectus sheath and rectus fibers, and then the fascia was sewn tightly with Prolene sutures.The catheter was grasped with a hemostat and pulled through the exit site incision. The wound was closed with silk sutures.  **Hospitalization time(day):28.0± 14.3** |
| Voss et al.2012 | Laparoscopic insertion of a PD catheter is performed under general anesthesia. The abdomen is insufflated and several small incisions are made lateral to the umbilicus. A needle is inserted with the establishment of pneumoperitoneum. A 5-mm trocar is inserted under direct visualization and a second 5-mm trocar is inserted just on the left lateral midline below the umbilicus, aiming towards the pelvis. The catheter is then threaded through the trocar and the cuff is pulled back so that it lays within the abdominal wall musculature. The catheter is then tunneled subcutaneously so it exits at the site of the original 5-mm trocar placement. The catheter is flushed to assess appropriate placement, and the skin sites are closed with interrupted suture.  **Hospitalization time(day):30.7±18.3** | Laparoscopic catheter insertions were performed by one credentialed specialist surgeon.(Operational specifics were not disclosed.)  **Hospitalization time(day):31.4±21.3** |  |
| Chula et al.2013 | Percutaneous insertion guided by radioscopy was performed using a technique developed by our own institution, which was based on the percutaneous insertion, using a trocar device, guided by radioscopy, which ensured to the operator that the catheter was correctly placed in the peritoneal cavity. |  | NA |
| Maher et al.2014 | Radiologic procedures were performed or assisted by four senior interventional.The technique used in the present study has been briefly described previously and is described in more detail later. The patient’s skin area was marked preoperatively for beltline and the proposed catheter tunnel exit site, prepared with povidone iodine 10%, and draped. An 18-gauge Hawkins–Akins needle (Cook, Bloomington, Indiana) with a blunt trochar was used to obtain access into the peritoneal cavity. The needle was inserted 2 cm inferiorly and laterally to the umbilicus and angled at a maximum of 451 to vertical and toward the contralateral iliac fossa from the exit site and puncture site. Access to the peritoneal cavity was confirmed by fluoroscopic screening of the injection of a small volume of iodinated contrast medium in the peritoneal cavity. A 0.035-inch super-stiff Amplatz guide wire (Boston Scientific, Natick, assachusetts) was inserted and coiled deep in the pelvis. Under fluoroscopic guidance, serial dilations were performed to allow passage of a 16-gauge peel-away sheath (Cook) over the guide wire into the peritoneal cavity. The peritoneal catheter (Curl Catheter; Baxter, Deerfield, Illinois) was passed over the guide wire through the sheath and into the pelvis without the use of any additional coaxial or stiffening placement system. The catheter was placed such that the first cuff was at the level of the rectus muscle sheath or the immediately adjacent subcutaneous fat. The sheath was then removed, followed by the guide wire, while using fluoroscopy to ensure maintenance of good catheter position. The subcutaneous tunnel was formed after initial blunt dissection with forceps to form a small pocket for the second cuff. Depending on the marked exit site location, an 8–15-cm length of subcutaneous tissue and skin was infiltrated with 2% lignocaine. Care was taken to ensure that the tunnel approached the exit site vertically from above, thus creating an “n”-shaped curved tunnel. A curved stainless-steel tunneling device was passed from the initial peritoneal access point along the subcutaneous tissues and then through the exit site via a small skin incision. The catheter itself was then attached to the trailing end of the tunneling device and pulled through the subcutaneous tunnel and exit site along with the tunneling device, taking care to avoid kinking of the catheter and disruption of cuff positions. Catheter patency was confirmed with the instillation and free drainage of 1 L of PD fluid. Note was made of the volume and appearance of the effluent. The catheter was secured to the skin with a sterile plastic dressing (IV3000; Smith and Nephew, London, United Kingdom) over an absorbent dressing (Primapore; Smith and Nephew). No sutures were used to secure the catheter. The insertion site was closed with interrupted sutures by using 3–0 nonabsorbable material; sutures were routinely removed 7–10 days after the procedure. | Laparoscopic insertions were performed or assisted by three senior surgeons; The surgical catheter insertion technique has been described in detail previously. Under general anesthesia, pneumoperitoneum was achieved with a 10-mm Hasson port through a vertical umbilical incision, and two 5-mm ports were placed for diagnostic laparoscopy and instrumentation. The PD catheter (CurlCatheter; Baxter) was inserted such that the distal cuff was placed preperitoneally, with the catheter tip in the rectovesical pouch. Care was taken to ensure that the catheter followed a smooth curve to exit the abdomen in a lateral and downward direction. Postsurgical care was identical to that with the radiologic insertion technique. |  |
| Sun et al.2015 | The procedures were performed by one of three credentialed specialist interventional radiologists. Standard two-cuff PD catheters with coiled tips were used for all procedures using this technique.(Operational specifics were not disclosed.) | This insertion technique was performed by one of two credentialed surgeons using laparoscopy. All insertions were performed under general anaesthesia. All catheters inserted were standard two-cuff PD catheters with coiled tips.(Operational specifics were not disclosed.) |  |
| Ahmed et al.2018 | Radiologic PDC placement using fluoroscopy and ultrasound guidance has been previously described and was performed by three interventional radiologists and one interventional nephrologist each with at least 5 years of experience. A micropuncture set was used to access the peritoneum. The laparoscopic catheter insertion was performed by one surgeon with at least 5 years experience in placing PD catheters using this technique.(Operational specifics were not disclosed.) | NA |  |
| Bin Chen et al.2021 | The patient is placed in a supine position for the ultrasound-guided percutaneous Seldinger puncture method. A mark for the incision is made 8 to 13 cm above the pubic symphysis, 2 cm to the side. Routine disinfection and draping are performed. Local anesthesia is administered with 1% lidocaine. The skin is incised, and the anterior sheath of the rectus abdominis is separated. A small incision of about 2 mm is made in the sheath. The trocar with a stylet is inserted into the abdominal cavity at a 45-degree angle under ultrasound guidance. The stylet is removed. The guidewire is inserted into the abdominal cavity along the trocar. The trocar is then removed, and the dilator with a peel-away sheath is inserted into the abdominal cavity along the guidewire. The guidewire and dilator are then removed. The metallic wire is lubricated with sterile liquid paraffin and threaded into the peritoneal dialysis catheter, bent at 135 degrees. The raised end is directed downwards along the anterior abdominal wall. When the patient reports the sensation of urination, the peritoneal dialysis catheter is rotated 180 degrees. The distal end of the catheter in the abdominal cavity is then placed into the rectovesical or rectouterine pouch. The metallic wire is removed. Subsequent steps are the same as those for the catheter placement group under laparoscopy.  **Hospitalization time(day):9.2 ±2.6** | The method of laparoscopic-guided subcutaneous tunneling is used for preoperative bladder emptying before peritoneal dialysis catheterization. The patient is placed in a supine position, and under general anesthesia, a tracheal intubation is performed to establish pneumoperitoneum, with the pressure maintained at 3 to 5 mmHg (1 mmHg=0.133 kPa), as tolerated by the patient. A 10 mm Trmar (puncture cannula) is used for puncture, and the laparoscope is inserted. The skin is incised, and the anterior sheath of the rectus abdominis muscle is separated. A small incision of about 2 mm is made in the anterior sheath, and the dilator with a stripping sheath is inserted into the posterior sheath of the rectus abdominis muscle. Subcutaneous tunneling is performed approximately 7 to 8 mm above the posterior sheath, and the inner needle core is inserted to perforate the peritoneum. Once the dilator with the stripping sheath enters the abdominal cavity, the needle core and dilator are sequentially withdrawn. The peritoneal dialysis catheter is inserted into the abdominal cavity along the stripping sheath, with the catheter tip located at the lowest part of the pelvic cavity (rectovesical or rectouterine fossa), and the polyester cuff is positioned just below the anterior sheath of the abdominal point muscle or within the muscle layer. The other end of the peritoneal dialysis catheter is connected to the tunnel needle. A subcutaneous tunnel is created, and the peritoneal dialysis external tube system is connected. Physiological saline is injected to test the patency of the catheter.  **Hospitalization time(day): 9.8 ±3.4** | Adopting the conventional open surgical method, the affected breast is placed in a supine position. The anesthesia method and incision location are the same as those for the percutaneous puncture group. The skin and subcutaneous tissue are longitudinally incised up to the anterior sheath of the rectus abdominis muscle (3 ~ 5 cm). The rectus abdominis muscle is bluntly separated up to the posterior sheath and peritoneum. The peritoneum is incised, and the size should be just large enough to allow the passage of a standard I'enchoff coiled tube, followed by purse-string suture. The peritoneal dialysis catheter is inserted into the abdominal cavity (the insertion method is the same as for the percutaneous puncture group): the purse-string is tied. The subsequent steps are the same as for the laparoscopic catheter insertion group.  **Hospitalization time(day):10.7 ±3.2** |
| Yibo Ma et al.2021 | Under the guidance of ultrasound, the Veress needle enters the abdominal cavity from the anterior sheath of rectus abdominis. Normal saline was injected to ensure that there was no obstruction. The guidewire was placed in the peritoneal fluid above the bladder. The Veress needle was removed, and a dilator was placed along the guidewire to dilate the anterior sheath of rectus abdominis until the peritoneum. Normal saline was injected again to ensure a smooth flow. The avulsion sheath with the core was inserted along the guidewire, and the peritoneal dialysis catheter was placed along the avulsion sheath. The sheath was torn from both sides until all parts lower than the polyester sheath in the peritoneal dialysis catheter entered. The peritoneal fluid drainage was unobstructed. |  | The traditional open surgery method was operated as follows: a 3–5 cm incision was cut on the skin and subcutaneous tissue under local infiltration anesthesia, the anterior sheath of rectus abdominis was cut longitudinally, and the rectus abdominis was dissociated bluntly to expose the posterior sheath of rectus abdominis. A small incision was cut in the posterior sheath, and the peritoneal dialysis catheter was placed in the abdominal cavity, uterus, and rectal lacuna under the guidance of guidewire. Seldinger technique was to use the Tenckhoff trocar, guidewire, and sheath system for operation. The catheter was inserted into the abdominal cavity without direct vision, and the deep polyester sheath was placed only outside the abdominal muscle tissue. |
| Zhen Li et al.2022 | The procedure was performed by a nephrologist referring to our modified technique which we reported earlier, incorporating ultrasound guidance and the use of a multifunctional cystostomy paracentesis trocar for percutaneous puncture. The  multifunctional cystostomy paracentesis trocar component has integrated functions of sharp-headed trocar core puncture, blunt-headed trocar core guidance, and semiring outer sheath blunt dilation by pulling out the built-in trocar core.The 18F multifunctional cystostomy trocar was rotated left and right slowly and stabbed into the abdominal cavity under ultrasound monitoring. The sharp-headed trocar core was replaced by the blunt-headed trocar core after breaking through the abdominal  wall, and insertion continued in the direction of the vesicorectal fossa (or rectouterine fossa). The blunt-head trocar core was pulled out after reaching the target area. The guidewire and catheter are placed inserted into the pelvic cavity through the outer sheath of the trocar and move it to the Douglas fossa guided by ultrasound. Liquid entering the vesicorectal pouch (or rectouterine pouch) concomitant with catheter injection of normal saline was observed under ultrasound to confirm correct catheter tip placement. |  | The position of the catheter deep cuff and exit site were marked before surgery. A PDC of appropriate length was selected according to the patient’s body type. Using a conventional disinfection towel, a longitudinal incision was made through the skin  and subcutaneous tissue to expose the anterior sheath of the rectus abdominis under local infiltration anesthesia. The rectus abdominis was passively separated to expose the posterior sheath, and a small opening was cut to allow the PDC to pass  through to the peritoneum. Following purse-string suture, the PDC end was moved into the cesicorectal fossa (or uterorectal fossa) through a small incision using a stainless steel guide wire. The proper placement was confirmed by linear saline  outflow upon injection. The pouch was then ligated, and after ensuring that there was no leakage around the PDC, the inner polyester sleeve of the catheter was embedded into the anterior sheath of the rectus abdominis. The anterior sheath of the rectus abdominis was then sutured. A subcutaneous tunnel was established and externally connected via a titanium joint and a short tube. Finally, subcutaneous tissue and skin were sutured. |
| Obaid et al.2023 | NA | NA | NA |
| Zheng et al.2023 | NA | NA |  |
| Note.—IGPC: Image-guided percutaneous catheterization; DVC: direct visualization catheterization; NA: no data | | | |
