# Supplementary material for: Comparative outcomes of image-guided percutaneous catheterization versus direct visualization catheterization for peritoneal dialysis: A meta-analysis
Source: PLoS One. 2025 Jul 7;20(7):e0325600. doi: 10.1371/journal.pone.0325600 (PMC12233245; doi:10.1371/journal.pone.0325600)

1. Forest plot of sensitivity analysis for infectious complications (total of ten)


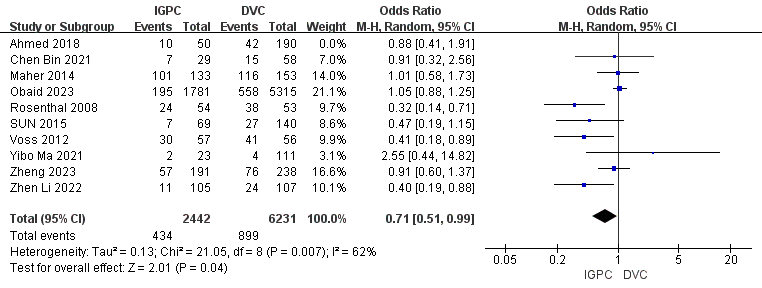


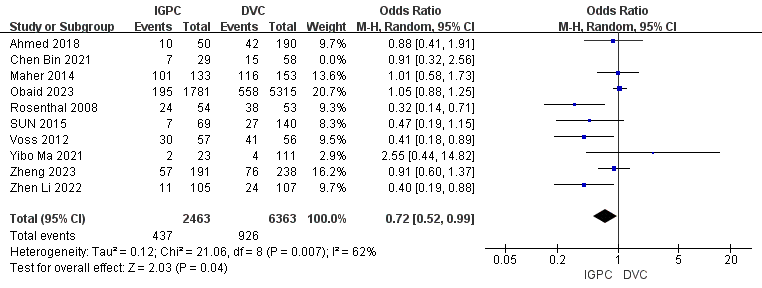


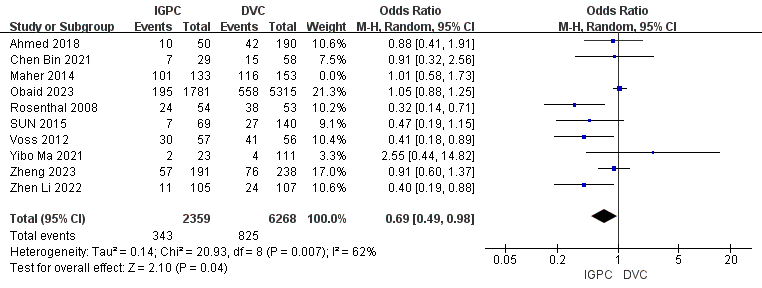


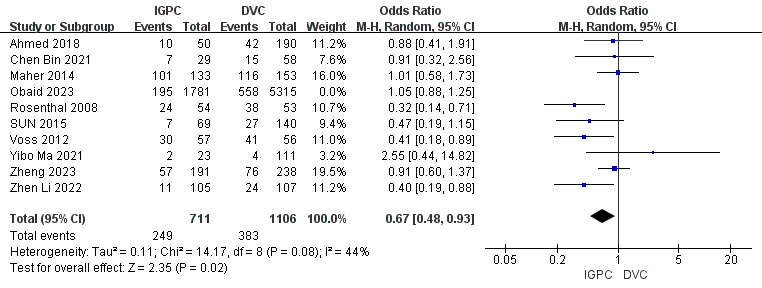


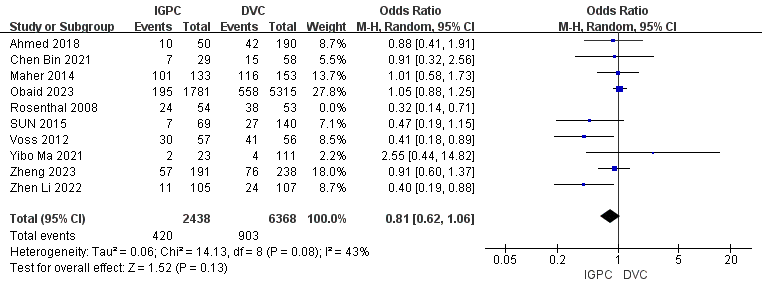


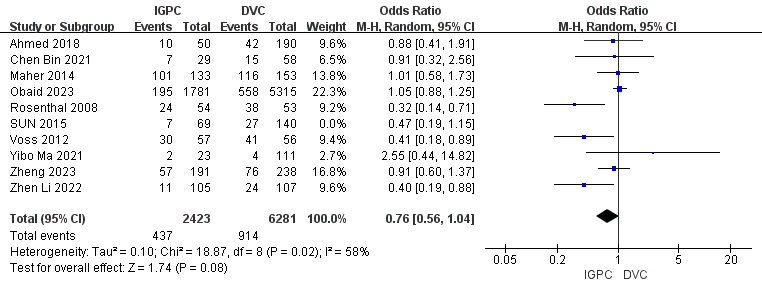


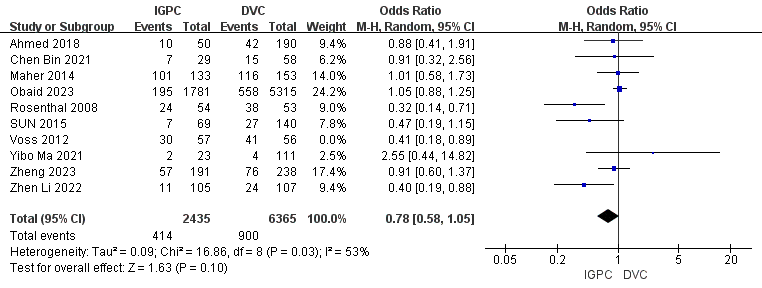


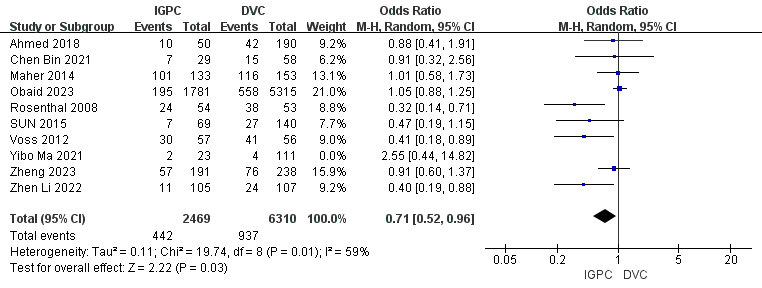


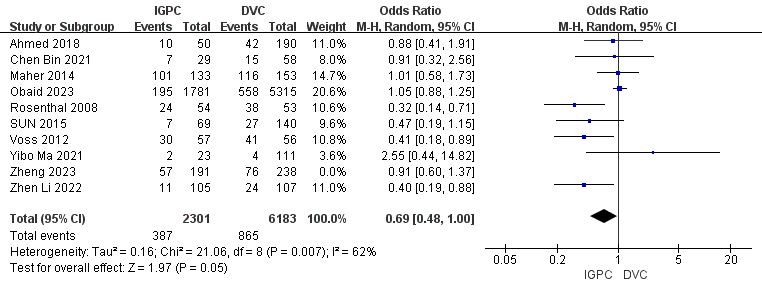


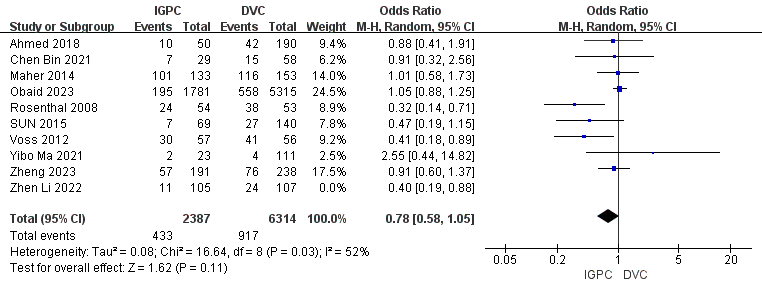


2.Forest plot of sensitivity analysis for mechanical complications (total of eleven)


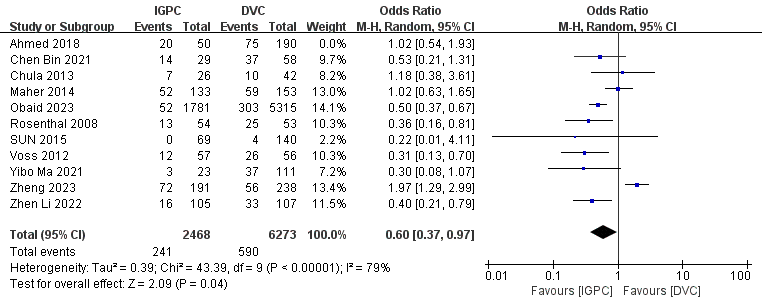


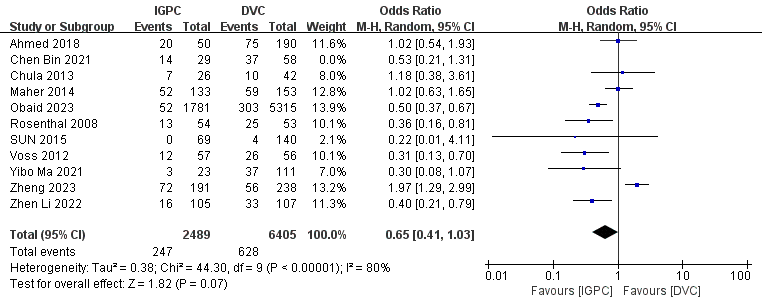


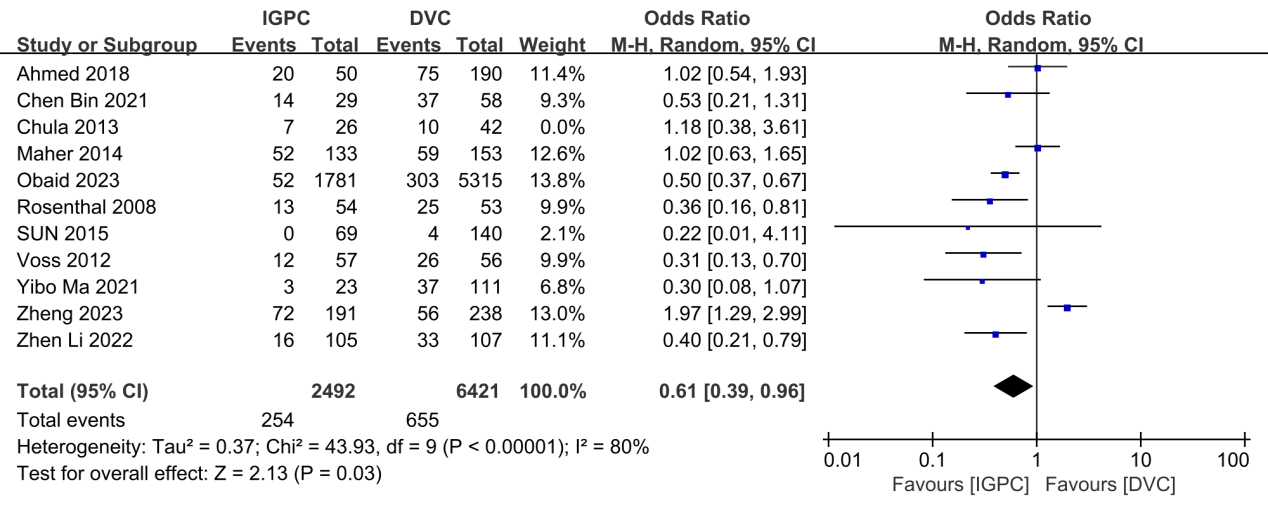


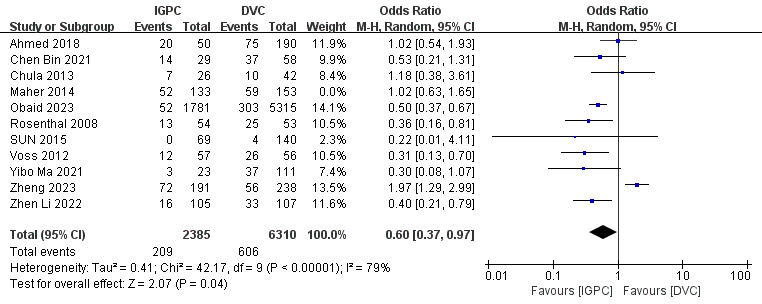


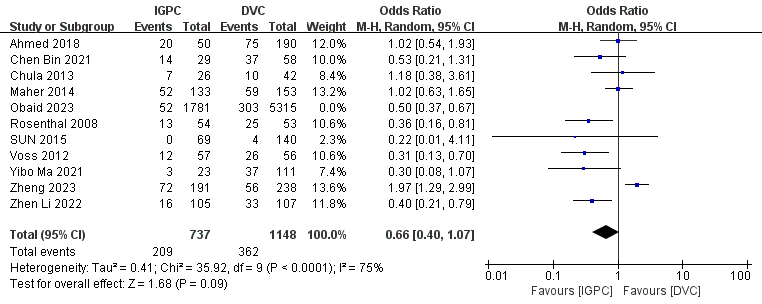


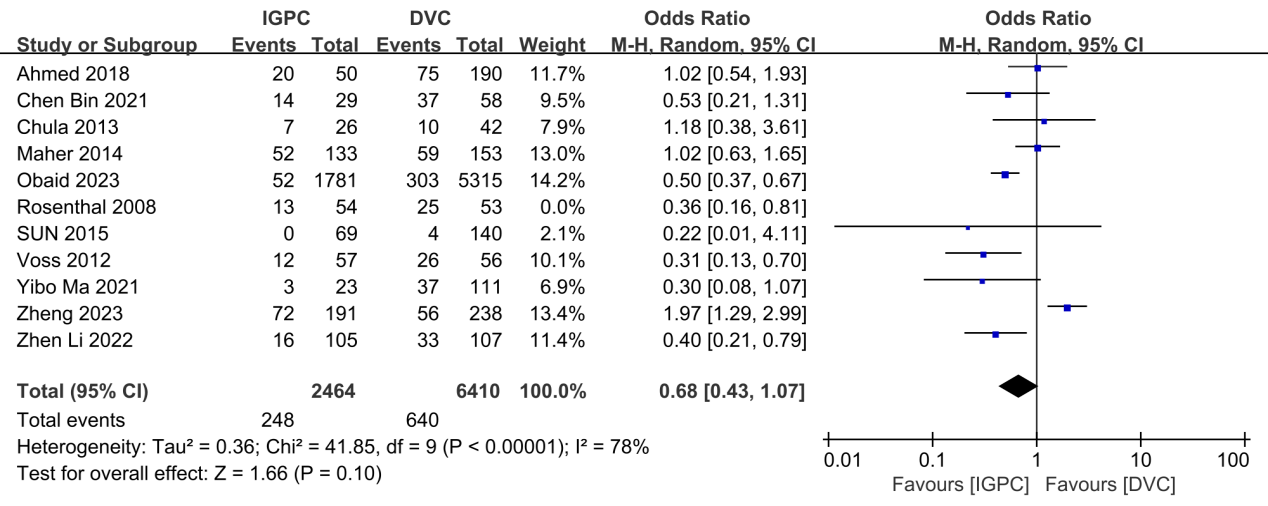


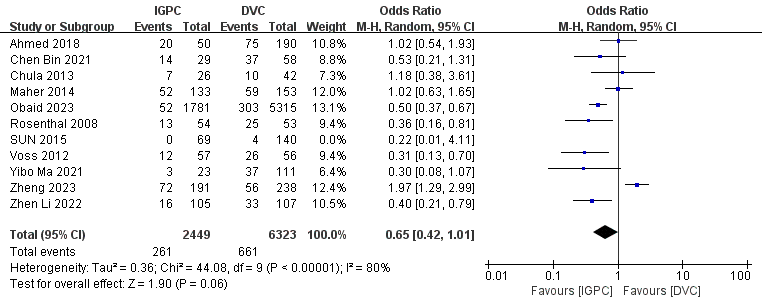


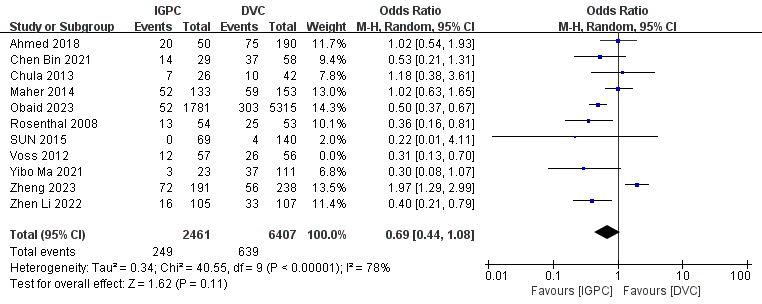


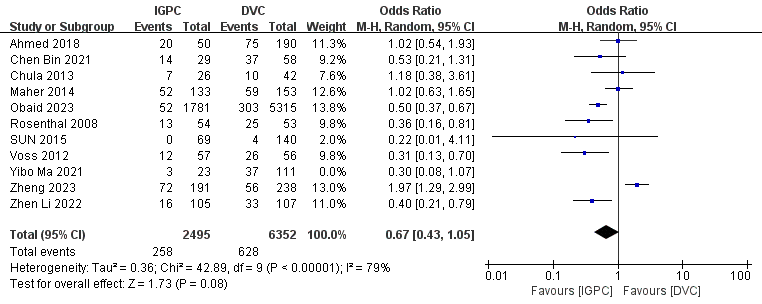


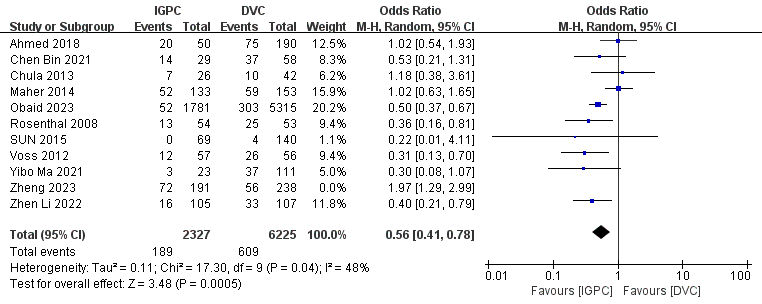


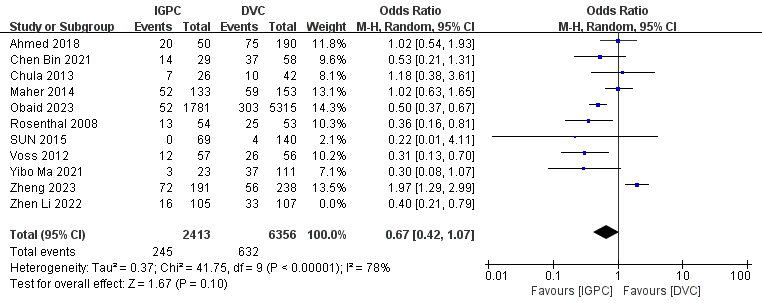


3.Forest plot of sensitivity analysis for one-year PD catheter survival rate (total of six)


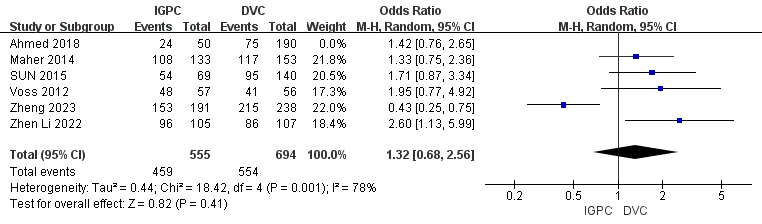


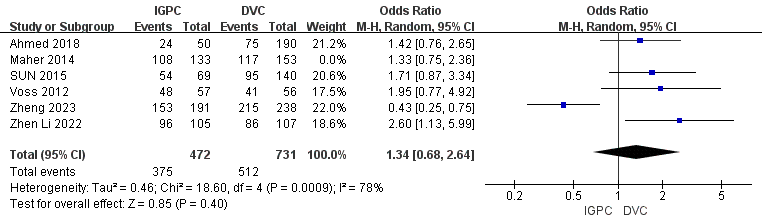


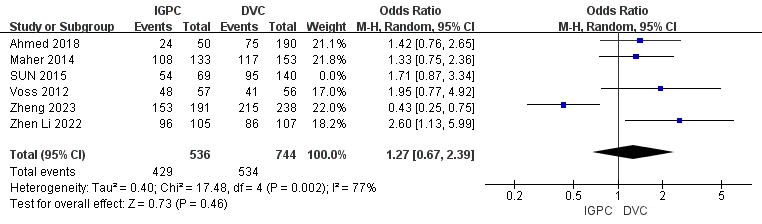


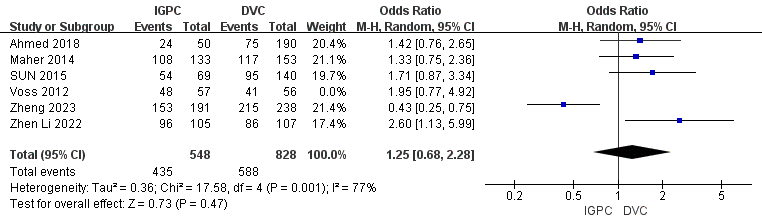


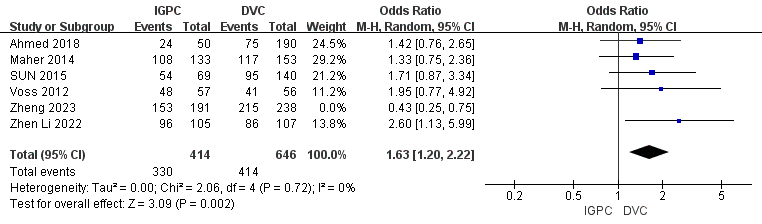

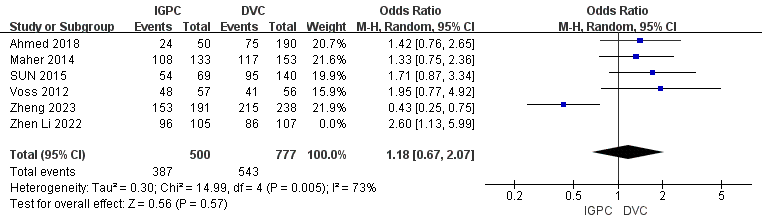


3.1. Sensitivity analysis forest plot for one-year PD catheter survival rate after removing the literature (Zheng 2023) (total of five).


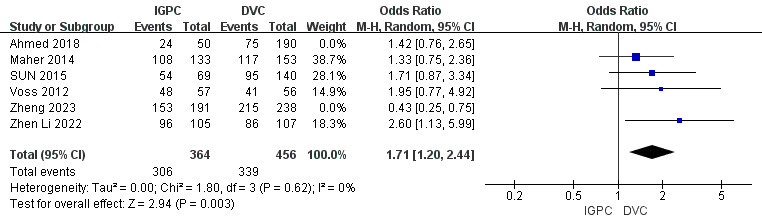

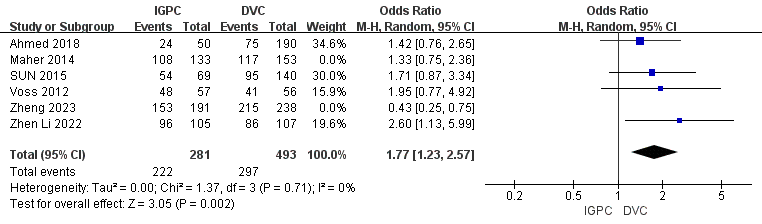


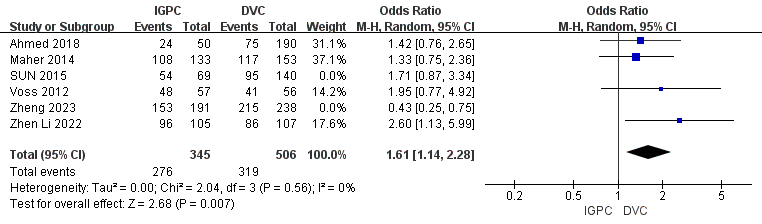


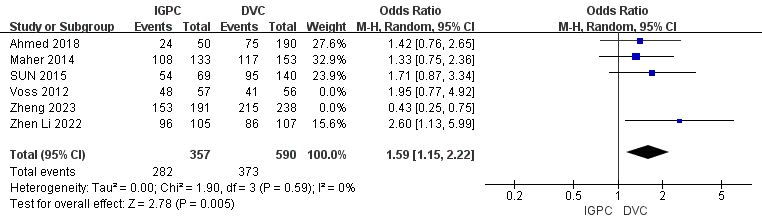

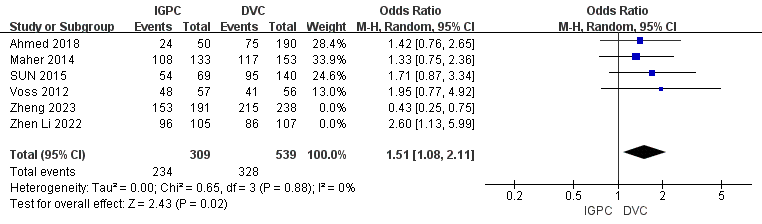


1. Forest plot of sensitivity analysis for catheter removal rates due to infection and mechanical complications (total of six).


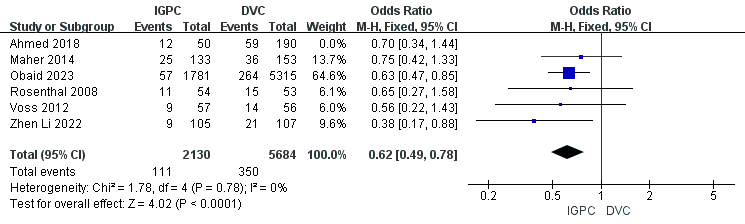


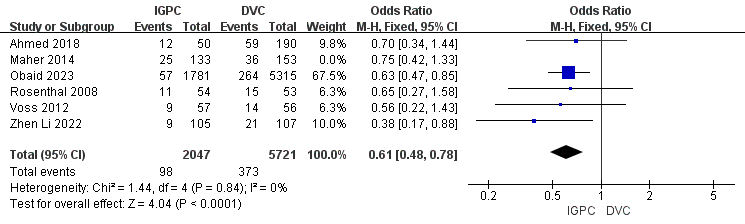

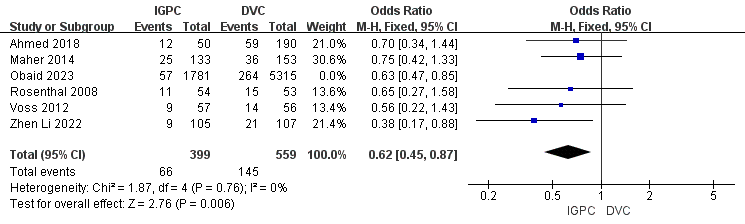

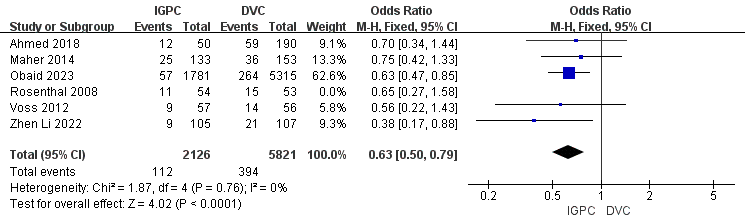

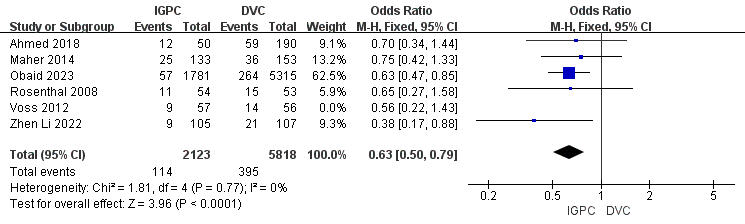

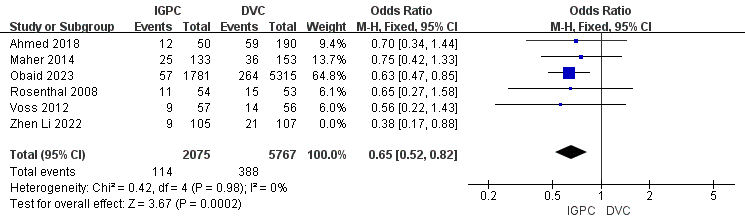

Supplement: S6 Text — (DOCX) [file pone.0325600.s006.docx]
