# Supplementary material for: Comparative outcomes of image-guided percutaneous catheterization versus direct visualization catheterization for peritoneal dialysis: A meta-analysis
Source: PLoS One. 2025 Jul 7;20(7):e0325600. doi: 10.1371/journal.pone.0325600 (PMC12233245; doi:10.1371/journal.pone.0325600)

**1.Forest plot of subgroup analysis for infectious complications:**


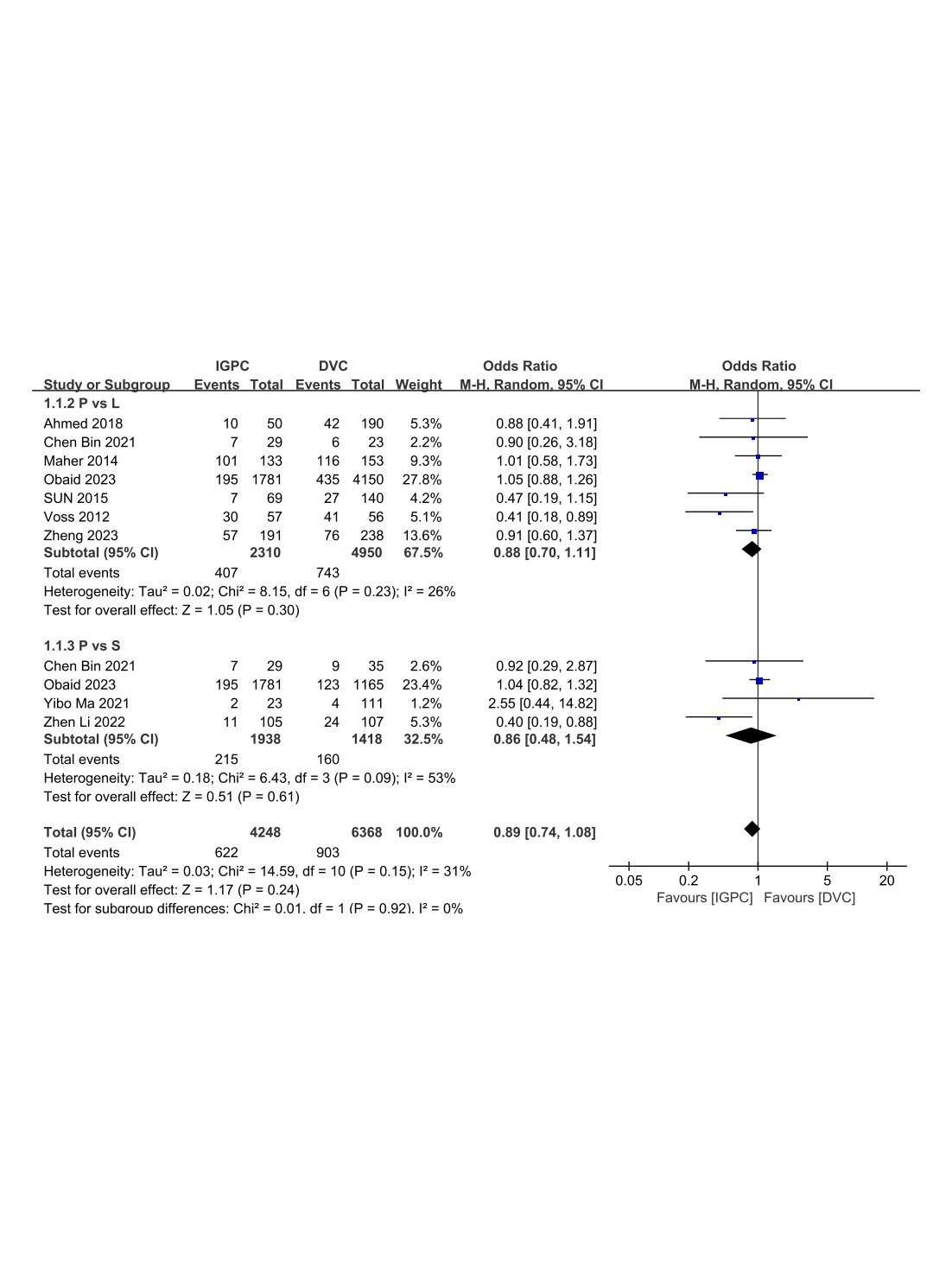


**2.Forest plot of subgroup analysis for mechanical complications:**


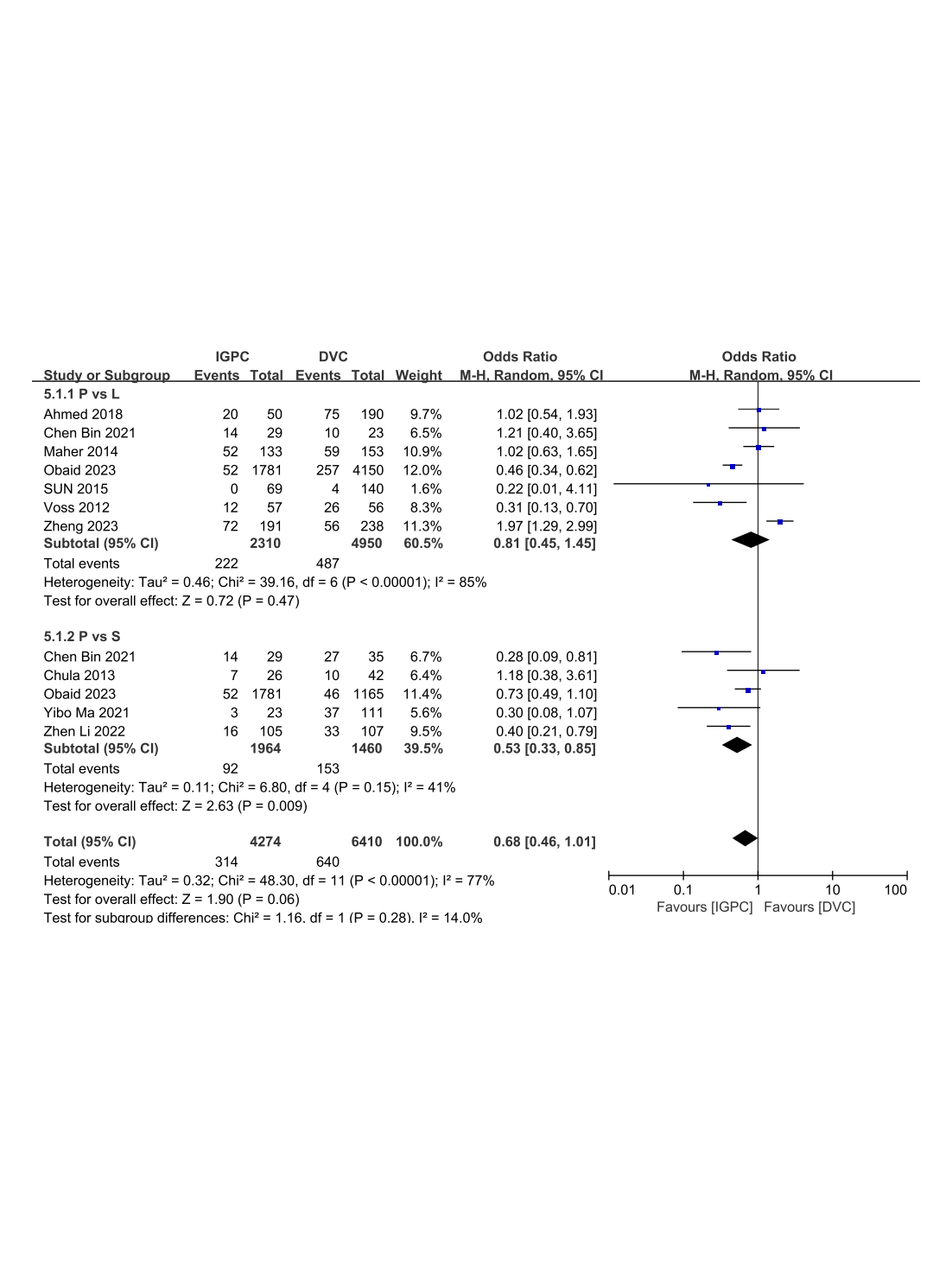


**3.Forest plot of subgroup analysis for one-year PD catheter survival:**


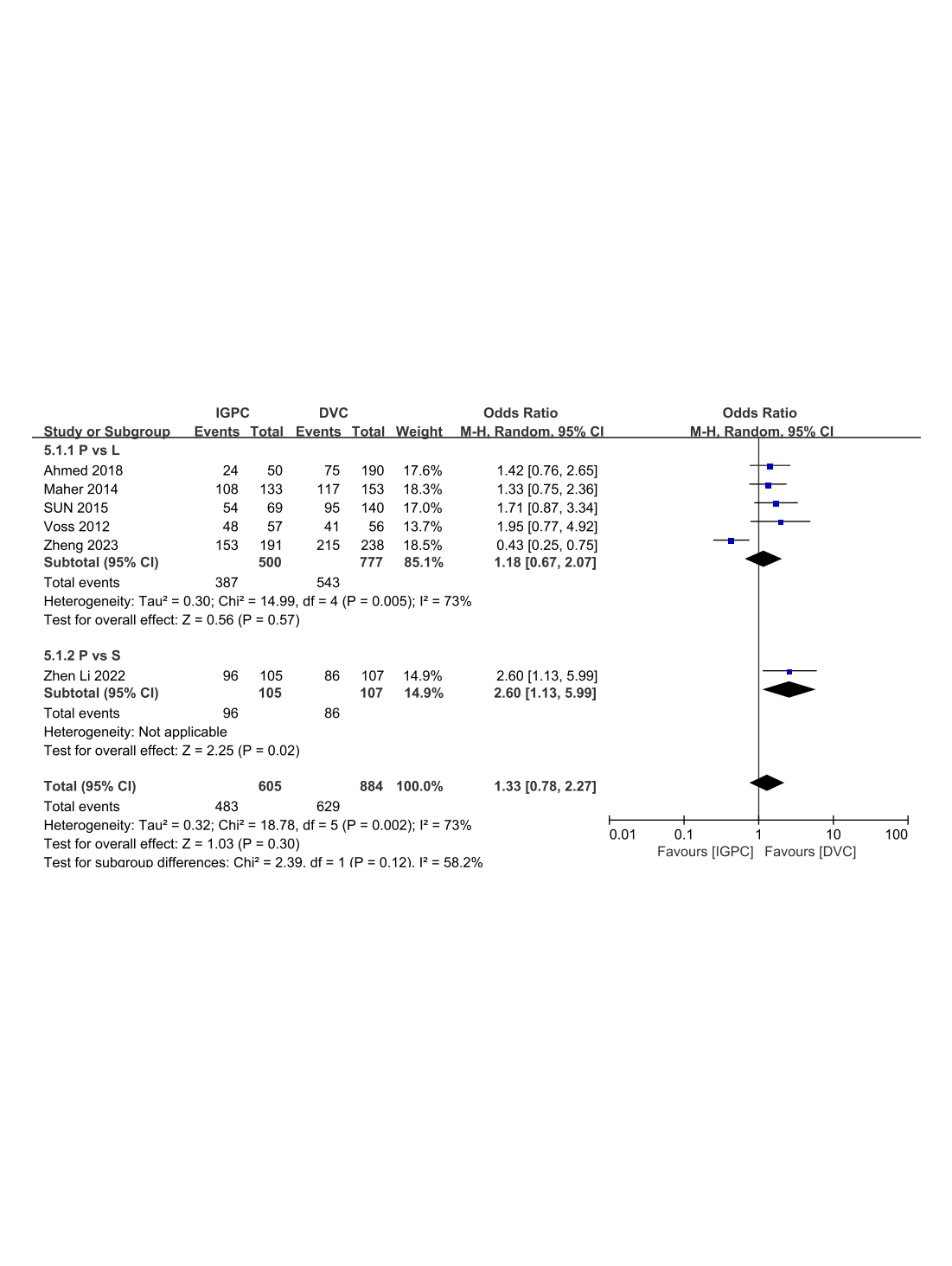


**4.Forest plot of subgroup analysis for catheter removal:**


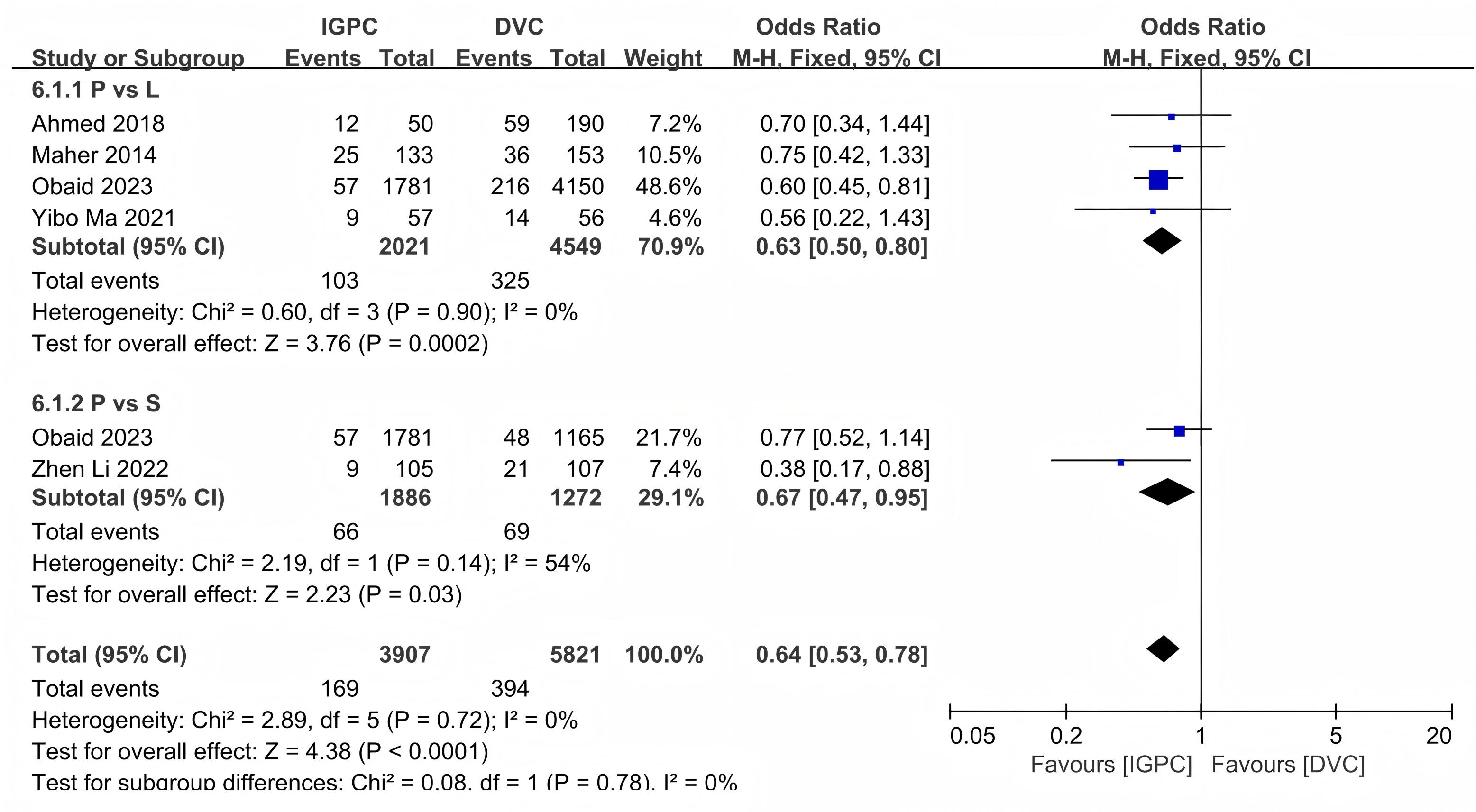

Supplement: S7 Text — (DOCX) [file pone.0325600.s007.docx]
