# Supplementary material for: Comparative outcomes of image-guided percutaneous catheterization versus direct visualization catheterization for peritoneal dialysis: A meta-analysis
Source: PLoS One. 2025 Jul 7;20(7):e0325600. doi: 10.1371/journal.pone.0325600 (PMC12233245; doi:10.1371/journal.pone.0325600)
Supplement: Attachment — Submitted filename: PLOS ONE REVIEW.docx [file pone.0325600.s009.docx]

Review on a manuscript titled: Comparative outcomes of image-guided percutaneous catheterization versus direct visualization catheterization for peritoneal dialysis: a meta-analysis

1. Title okay and other preliminaries,
   a. Change word catheterisation to catheterization, visualisation to visualization
   b. Other parts are okay, but authors may consider direct visualization as one of the key words
   c. All keywords must be written in a uniform way
   d. Abstract is relevant to the study
   e. Conflict of interest and financial disclosure are declared
2. Introduction
   a. Generally, well written and argued and the problem is apparent and concise)
   b. Sentence 35 ( …. Rate of up to 100,000 per …..) need completion
   c. Sentences 72-73 need revision, word therefore require completion
3. Search strategy (2.5) line 113 words ‘findings will be resolved’ should be changed to ‘revolved’
4. Analysis (2.6), well summarized, but generally difficult for non-scholars to understand, a more simplified language would make the section more understandable to most readers
5. Results (3),
   a. Lines 133-136 the description of the flow chart is not clear. The description articles were sieved from 970 to 620 is clear, however it is obscure thereafter. A clear picture of what was done from 620 to 11 should be added. It is noted however the flow chart is very clear on this.
   b. The rest of the result section appear well written
6. Discussion
   a. Generally, well written, but
   b. Paragraph one of the discussion parts appears a repetition of the introductory section than introducing the discussion
   c. The discussion is generally skewed towards literature review than discussing the interpretation, limitation and implication of study findings as proposed by PRISMA checklist, authors are advised to rewrite the discussion
   d. The discussion on blind insertion of the catheter in the last paragraph on infection complication of the discussion (230-232) is out of scope of this study. This review was on image guided peritoneal catheter insertion vs direct vision (laparoscopic and open).
   e. Discussion on mechanical complications lines 234-239 too on based on a topic which was reviewed in the current meta-analysis. Authors are advised to adhere to PRISMA checklist
   f. On the discussion on catheter removal line 252, there a typo (catheter ing) which need to be corrected,
   g. Line 262 words ‘markedly inferior to’ may be replaced by words ‘less frequent ..’
   h. Line 263 last sentence ‘IGPC demonstrated the advantage of visualization, resulting in decreased rates of infection and mechanical problems’ is misleading and may not be originating from this review
   i. The study limitations at the end of the discussion
7. Conclusion
   a. Well written, but hidden within the discussion texts
   b. Conclusion is informed by review findings
8. Funding – declared as required, authors should be put word ‘and’ before the last sentence
9. References and attachments
   a. References are according to journal, but reference 17 may not be relevant
   b. All attachments are available
