# Supplementary material for: Comparative outcomes of image-guided percutaneous catheterization versus direct visualization catheterization for peritoneal dialysis: A meta-analysis
Source: PLoS One. 2025 Jul 7;20(7):e0325600. doi: 10.1371/journal.pone.0325600 (PMC12233245; doi:10.1371/journal.pone.0325600)
Supplement: Attachment — Submitted filename: Response_to_Reviewers_auresp_2.docx [file pone.0325600.s011.docx]

Response to reviewers and editors

Dear Editor,

We would like to thank the reviewers and editors again for the careful revisions proposed in our manuscript (Comparative outcomes of image-guided percutaneous catheterization versus direct visualisation catheterisation for peritoneal dialysis: a meta-analysis). These comments and suggestions have definitely helped us to improve the quality of our paper. The following is an additional explanation of the missing parts in the initial revision.

Responses to reviewers' comments:

If applicable, we recommend that you deposit your laboratory protocols in protocols.io to enhance the reproducibility of your results. Protocols.io assigns your protocol its own identifier (DOI) so that it can be cited independently in the future. For instructions see: https://journals.plos.org/plosone/s/submission-guidelines#loc-laboratory-protocols.

**Response:** First of all, thank you for your suggestion. We have already registered the study protocol on PROSPERO (number CRD42024606795), so we do not intend to store the lab protocol in protocols.io anymore.

Reviewer #6: The authors have answered almost all of my comments. There is still one question which they have not addressed yet: "The authors state that the articles included were most recently up to 7/16/24. However, the authors did not include when the earliest date of the studies included in this meta-analysis was published, which would be very helpful in interpreting these results. Obviously, over time, our perioperative infectious rates have improved with antibiotic and sterilisation technique improvements. In addition, technological improvements in the development of PD catheters have also significantly improved since their inception." I think inclusion of how early these studies have started will really help inform the reader about the outcomes seen in this study.

**Response:** Thank you for the reminder that this was an oversight on our part. The issue has been modified in the original text, as detailed on pages 17, 82-83, and 143-144 of the revised text.

Special thanks to you for your comments.

We sincerely thank the reviewers for the time and effort invested in evaluating our manuscript, and hope that the correction will meet with approval.

Once again, thank you very much for your comments and suggestions.

Sincerely,
Tingting Liao
Corresponding Author
The First Affiliated Hospital of Guangxi University of Science and Technology, Guangxi University of Science and Technology

Email: 202301405159@stdmail.gxust.edu.cn
